# Dual action of chromium-reducing and nitrogen-fixing *Bacillus megaterium*-ASNF3 for improved agro-rehabilitation of chromium-stressed soils

**DOI:** 10.1007/s13205-016-0443-5

**Published:** 2016-06-07

**Authors:** Sumaira Aslam, Ali Hussain, Javed Iqbal Qazi

**Affiliations:** 1 Department of Zoology, Government College Women University, Faisalabad, Pakistan; 2 Department of Wildlife & Ecology, University of Veterinary & Animal Sciences, Lahore, Pakistan; 3 Department of Zoology, University of the Punjab, Lahore, Pakistan

**Keywords:** Bio-fertilizer, Bioremediation, Chromium bio-reduction, Chromium-reducing nitrogen fixer, Nitrogenase production, Wheat seedling

## Abstract

We conducted a study for enhanced biological rehabilitation of chromium-contaminated soils using a chromium-reducing and nitrogen-fixing bacterial species (*Bacillus megaterium*-ASNF3). The bacterial species was isolated from a chromium-rich land area, characterized, and employed under optimum conditions for the treatment of artificially prepared chromium-rich soil. The bacterium reduced Cr(VI) up to 86 % in a 60-day trial of incubation in the soil bioreactor. The nitrogenase activity of the bacterium yielded up to 486 nmol of ethylene/mL/h after an incubation period of 40 days when it was optimally cultured in growth medium at neutral pH and 30 °C. Although the nitrogen-fixing ability of the bacterium reduced significantly in the presence of 1000 ppm of Cr(VI), yet, the bacterium was proved to be a potential bio-fertilizer for enhancing nitrogen contents of the contaminated soil even under the higher chromium stress, together with the metal reduction. In the biologically treated soil, higher values of wheat growth variables were achieved. Application of metal-resistant *B. megaterium*-ASNF3 in selected situations rendered chromium-laden soils arable with significant increment in crop-yield parameters.

## Introduction

Industrial activities and sewage sludge depositions have largely contributed to the spread of toxic metals in terrestrial and aquatic environments. The long-term depositions of metal-loaded effluents have transformed fertile land areas into non-arable lands. Agricultural activities on such lands would result in bioaccumulation of toxic metals in food chain (Lu et al. 2011; Liu et al. 2013). Wide industrial applications of Cr in textile, leather tanning, metal finishing, and inorganic chemicals’ manufacturing result into discharges of Cr-loaded effluents into the environment. The environmental and public health concerns have made the metal contaminations a highly attention seeking problem (Zayed and Terry 2003).

Stable and, thus, commonly occurring oxidation states of Cr are trivalent Cr(III) and hexavalent Cr(VI) species (Kotas and Stasicka 2000). Cr(VI) is the most toxic one, because it has a high oxidizing potential, solubility, and mobility across membranes in the living organisms and through the environment (Azmat and Javed 2011). The carcinogenic and mutagenic effects of Cr(VI) to all forms of life are well known (Dong et al. 2007; Peralta-Videa et al. 2009). While Cr(III) being water-insoluble is much less toxic and usually precipitates as hydroxides (Shanker et al. 2005; Gheju et al. 2009). Cr contaminations mostly rise in urban lands where industrial effluents and wastewaters are applied for irrigation purposes and pose serious challenges regarding toxicity to vegetation growing on it and ultimately to the consumers of that vegetation (Shanker et al. 2005; López-Luna et al. 2009; Mushtaq and Khan 2010).

The most widely used method for the removal of metal ions is chemical neutralization. Given that this method is expensive and can possibly create generation of secondary pollutants, its application can be problematic (Ihsanullah et al. 2016). Owing to high efficacy and low operational cost, the bioremedial strategies have been advocated, including studies based on the utilization of genetic potential of microorganisms, originally isolated from the contaminated environment (Thacker et al. 2006; Cetin et al. 2008; Martins et al. 2010; Hussain and Qazi 2016). The line of action has high likelihood of isolating microbial cultures which can successfully be augmented for remediating the contamination.

Different ex situ bioremediation efforts while amending the contaminated soils with organic-rich contents and/or bacterial inoculations have shown promising results (Jeyasingh and Philip 2005; Khan et al. 2010). With a comparable objective, the present study was designed to isolate a bacterium having the potential to fix N and reduce Cr simultaneously, from highly Cr-contaminated soil and to employ the bacterial species for enhanced and improved agro-rehabilitation of Cr-stressed soil.

## Materials and methods

### Media

The medium used for the isolation of N-fixing bacterium, preservation in the slants as well as optimization of N-ase activity, was selective N-free agar medium (composition in gram per liter: K_2_HPO_4_ 1.0; FeS0_4_ 0.05; CaCl_2_ 0.1; MgSO_4·_7H_2_O 0.2; Na_2_MoO_4_ 0.001; glucose 10; agar 15) as described by Benson (1994). The nutrient broth medium (Merck) was used for reviving the bacterial growth from the N-free selective agar slants and preparation of inoculum. It was comprised of gram per liter: meat extract 3 and peptone from meat 5.

### Isolation of bacteria

The seed bacteria were isolated from Cr-contaminated soil of the city Kasur, Pakistan. The sampling area had been receiving the tannery discharges for more than 50 years. The soil was sampled from 20 to 40 cm depth of the study area, and 1 g of it was suspended in 10 mL of sterile water. Then, 0.1 mL of its serial dilutions was spread on selective N-free agar medium plates. The plates were incubated at 30 °C for 72 h. Following the appearance of growth on the selective medium, colonies were processed for routine pure culturing procedures.

### Identification of the bacterial isolate

The selected N-fixing bacterial isolate was characterized morphologically and physico-chemically as described by Benson (1994). The isolate was ultimately identified by 16S rDNA sequencing. For the purpose, DNA of the bacterial isolate was extracted from the overnight nutrient broth grown culture. Primers 27f (5′-AGAGTTTGATCMTGGCTCAG-3′) and 1492r (5′-GGTTACCTTGTTACGACTT-3′) were used for PCR amplification of 16S rDNA. The reaction was performed in total volume of 50 µL containing 5-µL DNA extract, 5 µL each of 25-mM MgCl_2_, 1-mM dNTPs, 5-pmol forward and reverse primers, 2-U/mL DNA *Taq* polymerase, and 1 X *Taq* buffer. The PCR cycle with denaturation for 3 min at 94 °C following 35 cycles of denaturation for 30 s at 95 °C, annealing step of 2 min at 60 °C and 1 min extension at 72 °C with a final extension step of 30 min at 72 °C was run in a thermal cycler (Hamburg 22331, Germany). For visualizing PCR product, 5 μL of PCR suspension was electrophoresed on 1 % agarose gel stained with ethidium bromide in TAE buffer. Amplified bands of 1.5 kb were visualized under UV (Gel Doc, Bio-Rad Laboratories, USA) to check for integrity. The DNA bands were purified using Gene Purification Kit (Fermentas) following the manufacturer’s instructions. They were then got sequenced using Big Dye Terminator v3.1 cycle sequencing ready reactions (Macrogen, Korea) at the DNA Sequencing Facility, Korea. 16S rRNA gene sequences were assembled with phrap (version 0.990319). Homology searches were performed using BLAST (http://www.ncbi.nlm.nih.gov/BLAST/).

### Optimization of the bacterial growth and N-ase activity

The bacterial isolate was grown overnight in nutrient broth at 37 °C. The growth (0.1 mL) was inoculated to 20 mL of N-free broth having pH 7.0 ± 0.5 and incubated at 30 °C for 7 days. For the determination of N-ase activity, 10 % of air was removed from the head space of the culture vial with the help of a sterile syringe to generate room for the gas to be injected. Then, acetylene was injected into the head space of the bacterial culture vial and incubated for 2 h at room temperature. C_2_H_4_ produced under N-ase catalysis was assessed biochemically following the method proposed by Larue and Kurz (1973) and nmoles of C_2_H_4_/mL/h were determined. One milliliter of the bacterial growth was harvested at prescribed post-incubation periods, and O.D. was recorded at 600 nm.

The bacterial isolate was inoculated and incubated at different conditions for determining enzyme-yield optima. For this purpose, the N-free broth at pH 7.0 ± 0.5, inoculated with 1 % of the bacterial growth, was incubated at 30 °C in daylight and dark for 10 days. For finding optimum temperature, the bacterial growth was incubated at 20, 25, 30, 37, and 45 °C for 10 days. In another set of experiments, N-free selective media having different initial pH values (5, 6, 7, 8, and 9) were inoculated with 1 % of the bacterial culture and incubated for 24 h at its respective temperature optima. After identifying optimum temperature and pH, the medium was inoculated with 1, 5, and 10 % of the bacterial culture and incubated for 10 days. While the effect of oxygen on N-nase activity was determined by incubating the bacterial culture at 120 rpm for aeration and without shaking for non-aeration at pre-determined temperature, initial pH, and inoculum optima.

### Effect of Cr(VI) stress on the bacterial growth and N-ase yield

The bacterial isolate was incubated in broth media spiked with 50, 100, 250, 500, and 1000 μg/mL of Cr(VI) and incubated at the optimum conditions. Growth and N-ase activity were assessed after 10 days post-incubation.

### Effects of C and N sources on the bacterial growth and N-ase yield

The bacterial isolate was further characterized for enhanced N-ase production with the incorporation of different C and N sources in the selective medium in the presence of 50 µg/mL of Cr(VI). For this purpose, 1 % (w/v) of sodium acetate, sodium citrate, sodium succinate, pyruvic acid, maltose, lactose, mannitol, galactose, xylose, and sorbitol were incorporated in the N-free media and incubated under optimum conditions. Similarly, 1 % (w/v) of urea, meat extract, yeast extract, tryptone, asparagine, peptone, sodium nitrate, ammonium tartrate, ammonium sulfate, and ammonium nitrate were added in the selected medium for observing effects of N-source on N-ase activity of the bacterium.

### Effect of incubation period on the bacterial growth and N-ase yield

The bacterium was inoculated in the optimal broth medium and incubated for 10, 20, 30, 40, and 50 days at the N-ase yield’s optima. At the termination of each experiment, analyses for growth and N-ase activity were performed.

### Soil collection and preparation for bioreactor

The soil was taken from the garden of University of the Punjab, Lahore, Pakistan and brought to the Microbial Biotechnology Laboratory, dried at 60 °C in oven for 24 h, and sieved with a sieve having mesh size of about 0.05 mm. The glass vials of 20-mL capacity with bottom diameter of 2.5 cm, an area of 56.91 cm^2^ and volume of 29.39 cm^3^ were filled with 10 g of the soil. The soil-filled vials were autoclaved and processed for bioremediation purpose excluding the one serving as a control bioreactor.

### Inoculum preparation

The bacterial culture was revived from slants to nutrient broth and incubated at 37 °C for 24 h. From this growth, inoculum for the soil bioreactor was prepared by incubating the bacterium in modified N-free medium at its growth optima (Benson 1994). In the modified N-free medium, galactose served as C source and peptone as N source. The bacterial culture was then centrifuged at 8000 rpm for 10 min, and the pellet was washed with sterile water. The cells’ suspensions in the water/modified medium of 0.2 O.D._600 nm_ were prepared to harvest in subsequent soil bioremediation experiments.

### Reduction of Cr(VI) in the soil bioreactors

Soil bioreactors entailing different nutrient conditions were prepared for studying bioremedial potential of the bacterium. Operating conditions for bioreactors are given in Table 1.

**Table 1:** Operational conditions of the soil bioreactors

Bioreactor type	Bacterial culture inoculated (0.2 O.D._600 nm_)	Nutrient supplements (mL)	dH_2_O (mL)	Cr(VI) (ppm)	Soil (g)	pH
Bioreactor 1	*Bacillus megaterium*-ASNF3	Nil	10	1000	10	7
Bioreactor 2	*Bacillus megaterium*-ASNF3	Ten modified N-free medium	Nil			
Control	Nil	Nil	10			

Ten milliliter bacterial suspensions in distilled water and modified medium were fed to the two categories of soil bioreactors contaminated with 1000 µg/mL of Cr(VI) in triplicates. The leachate above soil layer was sampled weekly to analyze Cr(VI) contents by the diphenylcarbazide method (Rehman et al. 2008). At the time of sampling, vials were shaken thoroughly for 2–3 min, and 0.1 mL of soil suspension was taken out for determining CFU of the bacterium. Then, soil particles were allowed to settle down and, 1.5 mL aliquot of liquid samples from vial was taken, centrifuged (5 min/8000 rpm), and the supernatant was used for analysis of Cr(VI). The experiments were terminated at the approach of constant level of Cr(VI), and the total Cr in liquid as well as solid phase of the bioreactors was thus determined with atomic absorption spectrophotometer (Perkin Elmer Analyst 400).

### Wheat growth in the treated soil

Eight-week bioremediated soils from each of the experimental sets mentioned in above section were assessed for wheat seed germination indices and the plant growth profiles. Eight-week treated soils (vials) of each experimental condition were processed in such a way that one set was autoclaved, while the second set was kept un-autoclaved.

The soil columns within the vials were plowed. The wheat seeds taken from a local market were sterilized with 0.5 % sodium hypochlorite solution for 1 min and embedded 1″ down the top soil. Another wide-mouthed and sterilized glass bottle was inverted over the soil-containing vial for maintaining aseptic conditions. Four seeds were sown in each vial. The vials with this setup were placed at 25 ± 1 °C for 10 days in daylight and night darkness for sprouting and growth. Seedlings’ growth was recorded at termination of the experiments after 10 days. The seedlings were recovered from the soil, and washed with distilled water and blot-dried. Weights of the fresh seedling as well as following drying at 80 °C for 24 h were recorded. Following the harvest, seedlings’ root and shoot lengths were measured. Chlorophyll ‘a’ and ‘b’ contents of seedlings were analyzed following Arnon (1949). Percent germination, moisture contents, seed vigor, and metal tolerance indices were calculated using the following formulae:

$${\text{Seed germination }}(\% ) = \frac{{{\text{Total no}}.\,{\text{of germinated seeds}}}}{{{\text{Total no}}.\,{\text{of seeds}}}} \times 1 0 0$$

$${\text{Seed vigour index (SVI) = Percent seed germination}} \times {\text{seedling size (cm)}}$$

$${\text{Moister contents }}(\% ){ = }\frac{{{\text{Fresh weight of seedling}} - {\text{dry weight of seedling}}}}{\text{Fresh weight of seedling}} \times 100$$

$${\text{Metal tolerance index = }}\frac{\text{Shoot/root lenght of treatment}}{\text{Shoot/root lenght of control}} \times 100$$

### Statistical analysis

The data were compared by analysis of variance (ANOVA) and student’s *t* test using Minitab-16 Software for determining significant difference at *P* < 0.05.

## Results and discussion

### Isolation and identification of the bacterium

Processing of the soil sampled from 40 cm below the surface from the study area yielded shiny-textured off-white, smooth, and convex colonies on the N-free agar medium plates. The bacterial isolate designated as ASNF3 was gram-positive, rod-shaped, motile and endospore former. The bacterium was found positive for catalase, nitrate, and Voges–Proskauer tests, and could hydrolyze casein, starch, and gelatin.

The BLAST analyses of the 16S rDNA sequences of the isolate ASNF3 showed 99 % similarity with *Bacillus megaterium* strain CCMM B583 (accession number: JN208059.1). The isolate was thus identified and designated as *Bacillus megaterium*-ASNF3. Its DNA sequence was submitted to NCBI database and has been allotted the accession number: KC527057.

### Optimization of the bacterial growth and N-ase activity

The bacterial growth and N-ase activity of *B. megaterium*-ASNF3 varied significantly under different incubation conditions (Table 2). The bacterium grew a bit better in the presence of light than under the dark incubation and showed higher N fixation. The N-ase activity of the bacterium persisted over a wide range of incubation temperatures (20–45 °C) with maximum yield of 1.7 nmol of C_2_H_4_/mL/h at 30 °C. An inoculum of 10 % of the bacterium expressed maximum N-ase activity (12.9 nmol C_2_H_4_/mL/h) with accompanying growth of 1.0 O.D._600 nm_, while anaerobic conditions were found favorable for higher N fixation. *Paenibacillus* and *Bacillus* species are well known to carry nifH genes which encode dinitrogenase reductase (Choo et al. 2003; Honga et al. 2012). Dark incubations lead to expression of niF gene for Fe-proteins (part of N-ase complex) and, hence, N-ase synthesis. Fe-proteins are modified during light phase and are speculated to be degraded by the proteases produced during light phase (Chow and Tabita 1994). Under low light levels, higher niF gene expression has also been reported by Steunou et al. (2008) in photosynthetic cyanobacteria and considered to be correlated with lower oxygen levels. He found higher nifH mRNA in the dark than under the light-incubation of *Synechococcus ecotype* which lead to ~6- and ~13-fold increment after 20 and 60 min, respectively. These studies support our findings of higher bacterial growth and N-ase activity under dark conditions. The higher bacterial growth brings about depletion in the oxygen level which may enhance the niF gene expression further and so the N-ase activity.

**Table 2:** Growth and nitrogen-fixing potential of *Bacillus megaterium*-ASNF3 cultivated in nitrogen free medium at different incubation conditions

Growth parameter	Incubation condition	Growth (O.D._600 nm_)	Nitrogenase activity (nmol of C_2_H_4_/mL/h)
Light conditions	Light	0.43 ± 0.01	2.04 ± 0.03
	Dark	0.38 ± 0.01	2.4 ± 0.03
Temperature (°C)	20	0.62^a^ ± 0.02	1.08^b^ ± 0.10
	25	0.32^b^ ± 0.03	1.7^a^ ± 0.12
	30	0.30^b^ ± 0.01	1.64^a^ ± 0.08
	37	0.21^c^ ± 0.01	1.16^bc^ ± 0.09
	45	0.17^c^ ± 0.02	0.77^c^ ± 0.03
pH	5	0.48^b^ ± 0.01	0.54^d^ ± 0.17
	6	0.41^b^ ± 0.02	3.25^c^ ± 0.17
	7	0.37^c^ ± 0.09	17.81^a^ ± 0.54
	8	0.45^b^ ± 0.03	6.77^b^ ± 0.24
	9	0.56^a^ ± 0.02	4.02^c^ ± 0.38
Inoculum size (%)	1	0.41^b^ ± 0.02	10.1^b^ ± 0.31
	5	0.54^b^ ± 0.03	12.42^a^ ± 0.36
	10	1.08^a^ ± 0.10	13.19^a^ ± 0.46
Aeration state	Aerated	0.63 ± 0.01	12.04 ± 0.32
	Non-aerated	0.73 ± 0.01	14.4 ± 0.36

Values are mean ± SE of three replicates

Those not sharing a common alphabet within a respective group are significantly different from each other

Single factor analysis of variance and Student’s *t* test. *P* ≤ 0.05

### Effect of Cr(VI) stress on the bacterial growth and N-ase yield

**Fig. 1:**
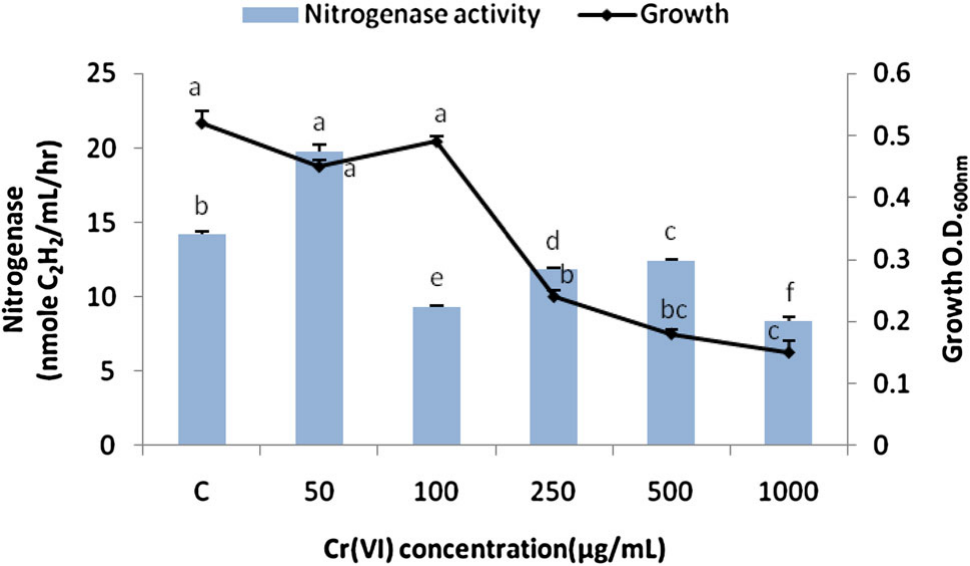
Effect of varying concentrations of Cr(VI) on the bacterial growth and nitrogenase yield following 10 days of incubation at the nitrogenase yield optima. *Bars* SE of respective means of three replicates. Different letters (*a*, *b*, *c*, *d*, *e*, *f*) indicate significant difference between different concentrations of Cr(VI) at *P* ≤ 0.05

The bacterium cultivated in N-free broth media spiked with 50, 100, 250, 500, and 1000 μg/mL of Cr(VI) at enzyme yield optima showed growths comparable with the control (≃0.5 O.D._600nm_) in the presence of 50 and 100 µg of Cr(VI)/mL (Fig. 1). The same parameters decreased down to 28 % in the presence higher Cr concentrations. The N-ase activity of *B. megaterium*-ASNF3 increased about 31 % in the broth with 50 µg/mL of Cr(VI) as compared with the control value, however, at higher levels of Cr it decreased down to 18 % in the media (Fig. 1).

Microorganisms under heavy metals’ stress produce various enzymes which mobilize heavy metals as well as certain chemicals, such as siderophores, one of the mechanisms by which *B. megaterium* alleviate heavy metal toxicity (Hu and Boyer 1996; Dimkpa et al. 2009; Santos et al. 2014). These mechanisms thus enable microorganisms to survive and perform their specific biogeochemical roles, such as N fixation under stress conditions.

### Effects of C and N sources on the bacterial growth and N-ase yield

**Fig. 2:**
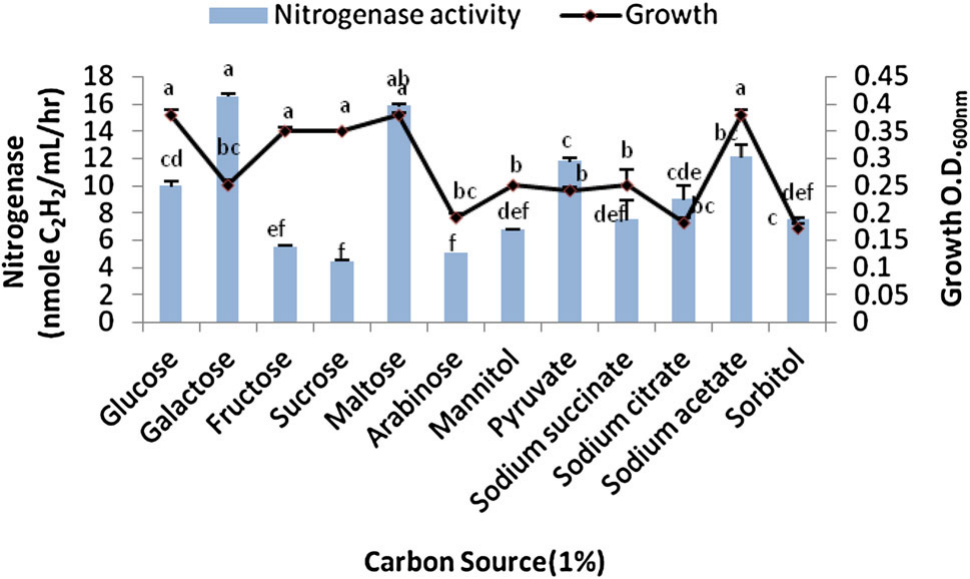
Effect of different carbon sources on the bacterial growth and nitrogenase yield. *Bars* SE of respective means of three replicates. Different letters (*a*, *b*, *c*, *d*, *e*, *f*) indicate significant difference between different carbon sources at *P* ≤ 0.05

Higher bacterial growth occurred in the presence of glucose, maltose, and the sodium acetate when compared with other C sources. Maximum acetylene reduction occurred in the galactose medium and yielded 16.55 nmol of C_2_H_4_/mL/h. This activity level was significantly higher as compared with other C sources. Maltose followed the galactose and produced sufficient N-ase (15.12 nmol of C_2_H_4_/mL/h). N-ase response to different C source is shown in Fig. 2.

**Fig. 3:**
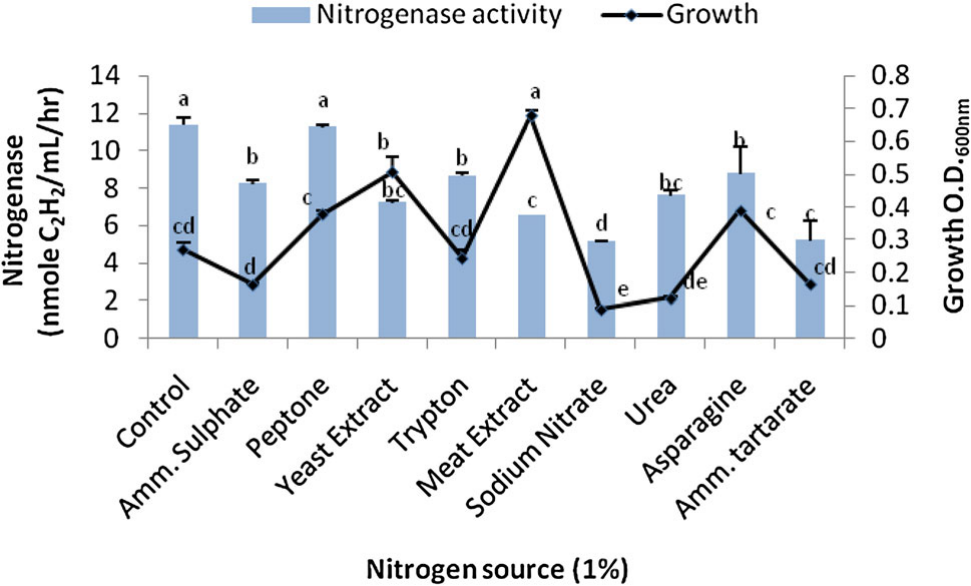
Effect of different nitrogen sources on the bacterial growth and nitrogenase yield. *Bars* SE of respective means of three replicates. Different letters (*a*, *b*, *c*, *d*, *e*) indicate significant difference between different nitrogen sources at *P* ≤ 0.05

The bacterial growth was enhanced significantly in the presence of N source as compared with N-free broth. Maximum cell densities appeared with meat extract and yeast extract with O.D._600 nm_ of 0.68 and 0.52, respectively. However, the addition of N source inhibited N-ase activity about 22–55 % when compared with N-free broth (11.35 nmol of C_2_H_4_/mL/h). Among different N sources, peptone appeared non-inhibitory to N-ase performance with the production of 11.26 nmol of C_2_H_4_/mL/h. These N-ase and growth responses are presented in Fig. 3. Rao and Venkateswarlu (1982) observed similar results with significant reductions in N-ase activity of *Azospirillum lipoferum* with inorganic N sources as compared with proteins and amino acids though growth was enhanced.

### Effect of incubation period on the bacterial growth and N-ase yield

**Fig. 4:**
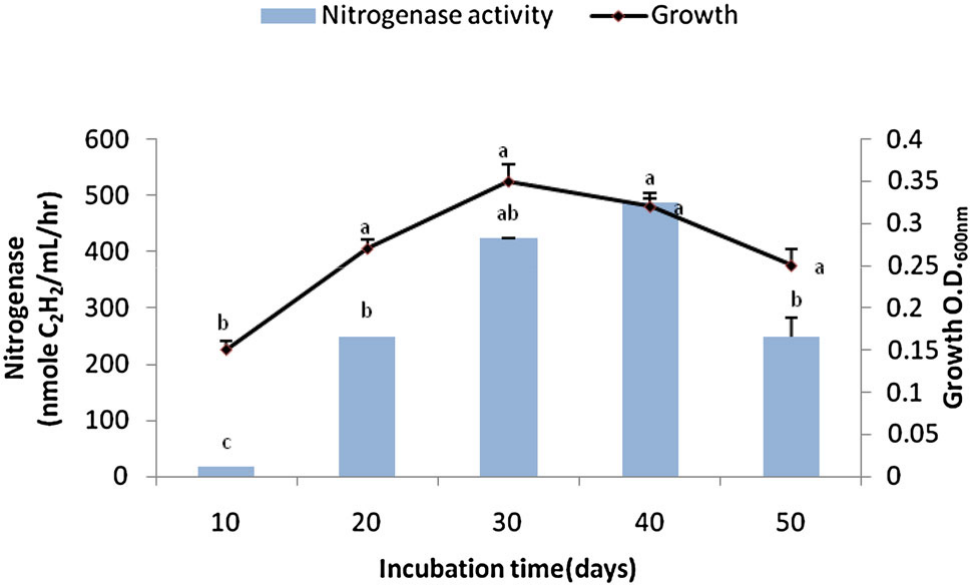
Bacterial growth and nitrogenase yield at different post-incubation periods. *Bars* SE of respective means of three replicates. Different letters (*a*, *b*, *c*) indicate significant difference between different incubation periods at *P* ≤ 0.05

The bacterial growth curve expressed two phases. A gentle progression from 0.15 cell density to 0.35 occurred during the 10 to 30 days post-incubation. This was followed by feeble decrements in growth down to 0.31 and 0.25 cell densities at 40 and 50 days post-incubation (Fig. 4). Accordingly, N-ase activity of *B. megaterium*-ASNF3 showed progressive increases till the 40th day of incubation, where 24.5-fold increment (418 nmol of C_2_H_4_/mL/h) was observed over the first sampling period, i.e., an incubation of 10 days (Fig. 4).

Many bacteria have been identified which could yield significant quantities of N-ase enzyme. Burgmann et al. (2004) reported N fixation up to 60 kg N/ha/year by free living N-fixing bacteria. *Bacillus* sp are also well-known diazotrophs which have the ability to fix atmospheric N. The N-ase yields of 25–292.5 nmol C_2_H_4_ mg protein/h have been recorded from *Bacillus* sp. in different studies (Ding et al. 2004; Ambrosini et al. 2016), but the effect of heavy metals particularly chromium on these yields under pure culture conditions is scarce. Thus, the findings of the present study declare the *B. megaterium*-ASNF3 a potential N-ase producer under chromium stress.

### Survival of the inoculants in the soil bioreactor

**Fig. 5:**
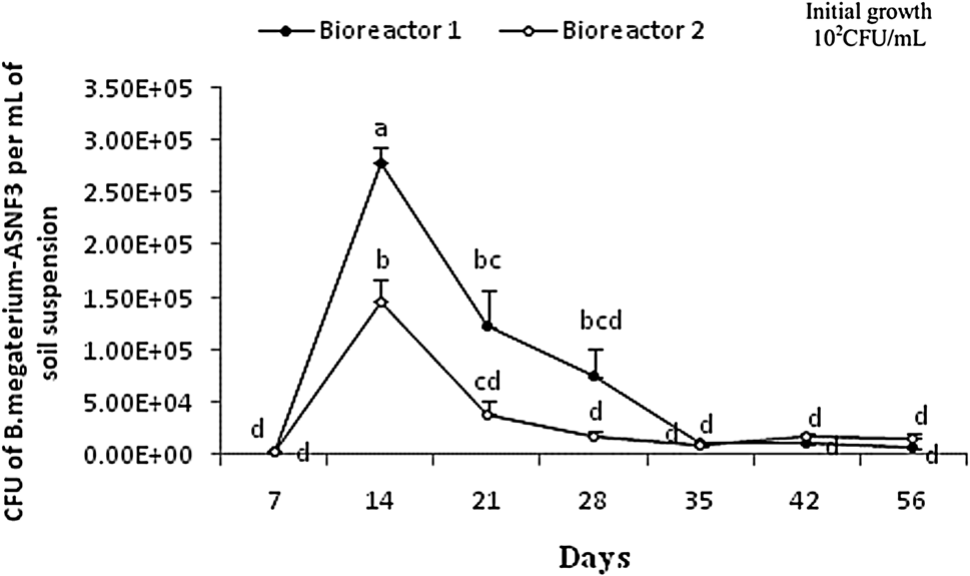
Growth trends of *B. megaterium*-ASNF3 in chromium-contaminated soil bioreactors under variable nutritional status. Different letters (*a*, *b*, *c*, *d*) indicate significant difference between different bioreactor conditions at *P* ≤ 0.05

The N-fixing rhizobacterium, *B. megaterium*-ASNF3, was employed as inoculant to remediate Cr-contaminated soil with an initial concentration of 1000 µg/mL of Cr(VI) under different nutritional conditions (Fig. 5).

The inocula of *B. megaterium*-ASNF3 in the soil bioreactor 2 responded negatively to the presence of additional nutrients as compared with the bioreactor 1. In the bioreactor 2, a rapid increase in CFU/mL up to 3 × 10^3^ appeared during the initial 7 days of incubation and reached the peak value of 1 × 10^5^ at 14th day of incubation. The same trend but with significantly higher CFU/mL of *B. megaterium*-ASNF3 at various sampling points was recorded for the bioreactor 1 (without additional nutrients). Gradual decrements in the growth after the peaks at 14th day post-inoculation were recorded for rest of the experimental period in both soil bioreactors 1 and 2 (Fig. 5).

Among different *Bacillus* spp. known for Cr reduction, *Bacillus megaterium* has its importance as plant growth promotor and for subsequent phytoremediation of heavy metals. It is a soil bacterium with heavy metals’ resistance capability and poses a great potential for their bioaccumulation (Cheung and Ji-Dong 2005; Wu et al. 2005).

### Reduction of Cr(VI) in the soil bioreactors

**Fig. 6:**
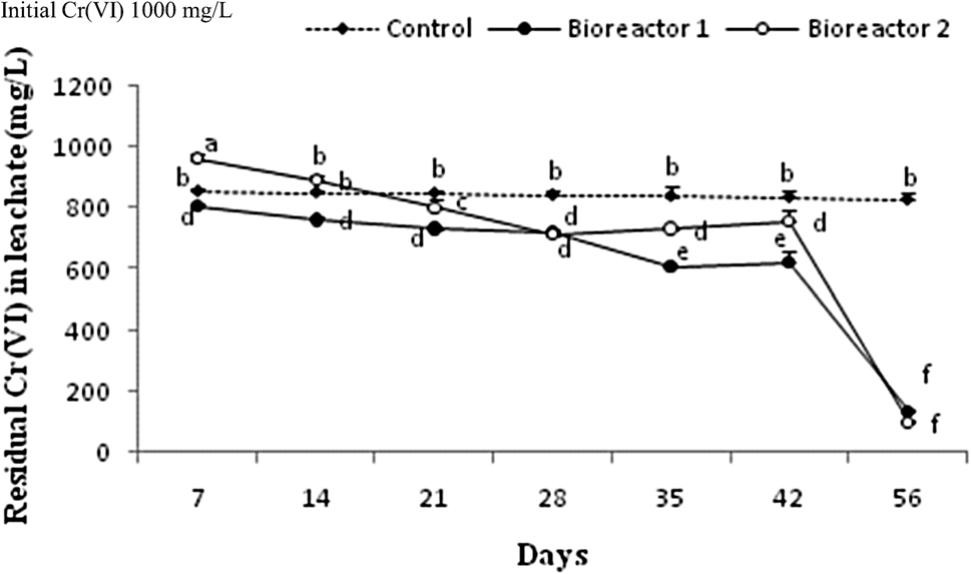
Effects of *B. megaterium*-ASNF3 inoculants on Cr(VI) reduction in chromium-contaminated soil bioreactors. Different letters (*a*, *b*, *c*, *d*, *e*, *f*) indicate significant difference between different bioreactor conditions at *P* ≤ 0.05

The *B. megaterium*-ASNF3 proved very efficient in reducing Cr(VI) in the soil bioreactors. The performance of the soil bioreactors is presented in Fig. 6. An appreciable abiotic reduction of Cr(VI) (about 15 %) of the total added was observed just after 7th day of incubation in control reactor, and thereafter, no further noticeable decrements were observed, while Cr(VI) reductions increased with increase in incubation period in experimental bioreactors 1 and 2. The bioaugmented soil without nutrient supplementation (bioreactor 1) appeared more efficient in reducing Cr than the nutrient-supplemented soil (bioreactor 2), except for the last sampling point (8th week). Decrease in the residual Cr(VI) was observed 1 through 6 weeks and ranged from 764 to 528 ppm in bioreactor 1, while the corresponding values of bioreactor 2 were 961 to 734 ppm, respectively. Cr(VI) dropped sharply during the last 15 days of incubation period and valued down to 134 and 99 ppm in bioreactor 1 and 2, respectively, which suggested that gradual change in pH of leachate from neutral to basic with increase in incubation time favored Cr(VI) reduction. These findings of the present study are consistent with those of Jeyasingh and Philip (2005) who reported that neutral to basic pH enhanced Cr(VI) reduction in biological treatment systems.

The rhizobial bacteria could survive metal-harsh conditions through different mechanisms of metal resistance, efflux, bioaccumulation, or bioreduction (Outten et al. 2000). It is also known that bacterial tolerance to metals vary according to the media conditions and composition (Rajkumar et al. 2005). The soil bacterium, *B. megaterium*-ASNF3 had a history of exposure to Cr contamination and thus exhibited significant Cr(VI) reduction potential under soil suspension conditions. Complete reduction of Cr(VI) was not observed in any of the two experimental soil bioreactors; however, significant Cr reductions up to 84 and 86 % were achieved in the bioreactors 1 and 2, respectively, render the bacterium a candidate of soil bioremediation. Many microorganisms indigenous to waste contaminated environments showed resilience to recalcitrant pollutants and, hence, revealed efficient bioremediation performances (Shakoori et al. 1999; Jeyasingh and Philip 2005). Cr-resistant and gram-positive bacteria in this regard have previously been successfully characterized for Cr reduction and implemented for bioremediation of soils (Shakoori et al. 1999; Kathiravan et al. 2011).

At the termination of experiment (60 days incubation period), mass balance of Cr was determined (Table 3). It was observed that higher fraction of total Cr was present in soil suspensions, while lesser mass was held in the soil. Following 8 weeks of incubation, Cr(VI) in soil bioreactors 1 and 2 suspensions could measure only up to 134 and 99.12 ppm, respectively. Another important observation was that total Cr bound to soils was higher in the nutritionally supplemented bioreactor 2. Bolan et al. (2003) reported that organic amendments in chromate-contaminated soils enhanced the organic bound fraction of the metal in soil and decreased soluble and exchangeable Cr fraction. A possible explanation could be the formation of more Cr(OH)_3_ under basic pH attained during detoxification mechanism which restricted its availability to the soil surface, and hence, its absorption and bioaccumulation in plants is controlled (Zhang et al. 2006). Surfaces of bacterial cells also serve as sites for specific electrochemical interactions with metals (Wang and Chen 2006), this effect rationale the differences in total added Cr (1000 ppm) in the bioreactors 1 and 2, and the one observed at end of the experiments. The metal adsorbed to bacterial surface might had become unavailable for the analyses. The N-ase activity of *B. megaterium*-ASNF3 at termination of the experiments appeared up to 14.6 and 21.8 nmol of C_2_H_4_/mL of soil suspension/hr in the bioreactors 1 and 2, respectively.

**Table 3:** Chromium mass balance and nitrogenase activity in soil bioreactors at termination of the experiment

Soil bioreactor	Initial Cr(VI)	Mass of Cr(VI) in soil suspension	Mass of total Cr in soil suspension	Mass of total Cr in soil	Total Cr	Percent reduction	nmol C_2_H_4_/mL/h	pH
Control	1000	829^a^ ± 21	660^c^ ± 32	340^a^ ± 9	1000^a^	17^b^ ± 0.5	ND	7.1^c^ ± 0.02
Bioreactor 1 (BmN_+ve_)		134^b^ ± 2	825^a^ ± 31	163^c^ ± 3	988.5^b^ ± 43	84^a^ ± 2	14.6 ± 0.9	7.5^b^ ± 0.03
Bioreactor 2 (BmN_−ve_)		99.12^c^ ± 4	682.75^b^ ± 23	290^b^ ± 5	972.75^c^ ± 51	86^a^ ± 3	21.8 ± 0.8	8.1^a^ ± 0.01

Values are mean ± SE of three replicates

Those not sharing a common alphabet within a respective group are significantly different from each other

Single factor analysis of variance and Student’s *t* test. *P* ≤ 0.05

The present results demonstrated that maximum growth and reductions were achieved at different incubation times and seemed not highly related. This trend is well justified by the studies of Viti et al. (2007) who reported that bacterial growth and chromate reduction are not always related and depend on carbon/energy source which decouple growth and Cr reduction. The above elaborated attributes of *B. megaterium*-ASNF3 render the isolate a study model for rehabilitating the metal-contaminated soils.

### Wheat growth in the treated soil

Wheat growth was greatly affected by varying soil treatments (Table 4). The soils treated with bacterial inoculations showed 100 % seed germination as compared with Cr-exposed control soil where seed germination dropped to 25 %. Susceptibility to Cr toxicity depends upon concentration of the metal and nature of the seeds used. For example, application of 500 ppm of Cr reduced 48 % seed germination of *Phaseolus vulgaris* (Parr and Taylor 1982) in contrast to 75 % reduction of wheat seeds in the present study. Jain et al. (2000) reported up to 57 % germination decline in sugarcane bud exposed to 80 ppm of Cr. Declines in seed germination indicate inhibitory attributes of Cr to particular enzymes’ activity involved in seed germination (Zeid 2001). Results of the present and earlier studies dictate for the usefulness of Cr-resistant bacterial inocula that are inhabitant of specific plant(s)’ rhizosphere for recovering agri-potentials of Cr-contaminated soils.

**Table 4:** Effect of *B. megaterium*-ASNF3 in different soil treatments on growth variables, seed vigor, metal tolerance, and chlorophyll contents of wheat seedling

Treatment code	Treatment	Seed germination (%)	Root length (cm)	Shoot length (cm)	Root/shoot ratio	Seedling height (cm)	Seedling fresh weight (mg/plant)	Seedling dry biomass (mg/plant)	Moisture contents (%)	Seed vigor index	Metal tolerance index	Chlorophyll a (mg/g)	Chlorophyll b (mg/g)
IC	Intact control	100	2.77^a^ ± 0.04	4.6^a^ ± 0.05	0.61^cde^ ± 0.01	11.21^a^ ± 0.43	170^a^ ± 3.5	147.5^ab^ ± 2.16	13.17^f^ ± 0.00	1121^a^ ± 43.3	100^a^ ± 0.00	2.81^a^ ± 0.02	0.55^fg^ ± 0.03
CrC	Chromium-spiked control	25	0.2^g^ ± 0.00	0.7^f^ ± 0.00	0.28^f^ ± 0.00	1.5^d^ ± 0.00	40^e^ ± 0.00	30^f^ ± 0.00	25^cde^ ± 0.00	37.5^d^ ± 1.00	7.2^g^ ± 0.00	1.2^e^ ± 0.01	0.21^h^ ± 0.01
ABmN_+ve_	Dead *B. megaterium*-ASNF3 + MVII medium	100	1.2^cd^ ± 0.1	1.4^bc^ ± 0.17	0.89^ab^ ± 0.09	3.7^bc^ ± 0.17	135^bc^ ± 5.59	85^de^ ± 5.59	37.29^bcd^ ± 1.55	370^bc^ ± 17.67	44.22^cd^ ± 3.9	1.26^d^ ± 0.04	0.72^bc^ ± 0.03
BBmN_+ve_	*B. megaterium*-ASNF3 + MVII medium	100	1.5^bc^ ± 0.07	1.4^b^ ± 0.09	1.09^a^ ± 0.09	3.8^bc^ ± 0.18	145^ab^ ± 5.59	107.5^cde^ ± 4.14	25.72^bcde^ ± 2.21	380^bc^ ± 18.37	55.05^bc^ ± 2.7	1.43^c^ ± 0.06	0.88^ab^ ± 0.00
ABmN_−ve_	Dead *B. megateriums*-ASNF3	100	0.73^f^ ± 0.02	1.32^cd^ ± 0.05	0.55^de^ ± 0.04	3.2^c^ ± 0.35	160^a^ ± 9.35	90^cde^ ± 6.12	42.3^ab^ ± 6.38	320^c^ ± 35.88	26.35^de^ ± 0.97	2.44^b^ ± 0.02	0.52^fg^ ± 0.02
B BmN_−ve_	*B. megateriums*-ASNF3	100	0.85^de^ ± 0.02	1.15^e^ ± 0.02	0.74^bcd^ ± 0.01	3.35^c^ ± 0.31	110^d^ ± 3.5	72.5^e^ ± 4.14	34.05^bcd^ ± 3.4	335^c^ ± 31.72	30.86^def^ ± 0.63	1.39^c^ ± 0.02	0.71^cde^ ± 0.03

Values are mean ± SE of three replicates

Those not sharing a common alphabet within a respective group are significantly different from each other

Two factor analysis of variance test. *P* ≤ 0.05

Wheat plants grown in un-autoclaved soils remediated with *B. megaterium*-ASNF3 expressed maximum root (1.5 cm) and shoot lengths (1.4 cm) which were 7.5-and 2-folds higher than the corresponding parameters of wheat seedling from CrC, respectively. These measurements of root and shoot were lower than those obtained from the wheat grown in IC (Cr un-amended and non-inoculated). CrC-loaded with 1000 µg/mL of Cr(VI) inhibited root growth significantly, while no shoot emergence was observed when compared with the IC. Inoculations of soils with *B. megaterium*-ASNF3 yielded 14 and 20 % higher root lengths of wheat plants in nutrient-supplemented and non-supplemented soils as compared with those cultivated in similarly treated but autoclaved soils. Since plants absorb nutrients through roots, accumulation of metals along with nutrient absorption occurs. It is also known that heavy metals could either affect physiological processes of the plants or completely inhibit their growth, while accumulation of metals in roots and bioaccumulation up to toxic levels in other plant parts affect overall growth and plant yields (Zayed et al. 1998; Ali et al. 2011).

Plants’ fresh and dry weights were also affected under varying soil treatment systems. The set of un-autoclaved soils initially remediated with cultures of *B. megaterium*-ASNF3 (ABmN−ve) without additional nutrients indicated up to 300 % increase in fresh weight of the seedlings than the CrC. While the levels of chlorophyll ‘a’ were high in comparison with chlorophyll ‘b’ in all treatments, chlorophyll ‘b’ showed marked increases over the IC in inoculated soils with biologically active treatments. Higher photosynthesis is requisite for production of organic molecules and plant biomass under Cr contamination (Bishnoi et al. 1993; Amin et al. 2013). These findings are in accordance with previous studies which demonstrated that decreases in dry biomass of plants are often result of Cr-toxic effects on chlorophyll contents inhibiting CO_2_ assimilation (Chatterjee and Chatterjee 2000; Nichols et al. 2000; Saygideger et al. 2013).

Although categories of the soils treated with *B. megaterium*-ASNF3 showed lower vigor when compared with the IC, a significant recovery in seedling vigor resulted when grew in bioremediated soils with up to 10-fold increase over those of the CrC. The metal tolerance index of the seedling was also higher in bioremediated soils as compared with the CrC. The seed vigor as well as metal tolerance indices as the important parameters for assessment of any crop’s yield influencing factor. Elaborated effects of *B. megaterium*-ASNF3 on seed vigor index (up to 830 % increase over CrC) prove the bacterium a potential candidate for obtaining healthy plant yields from the contaminated soil.

The N-fixing bacterial inoculations ameliorated the metal-induced stress in wheat and stimulated the plant growth as compared with the corresponding values obtained for the CrC. It is known that bacteria pose various mechanisms of metal detoxification, such as reduction, efflux, biosorption, and bioaccumulation. All these mechanisms ameliorate the toxic effects of metals. Cleaning of toxic metals from contaminated soils using such bacteria as biological inoculants could achieve land recovery for agricultural purposes (Ahluwalia and Goyal 2007; Khan et al. 2010). The seed germination and growth indices of wheat in this study underwent drastic inhibitions up to 75 % in the CrC as compared with the corresponding values obtained for the IC. However, the Cr-loaded soils yielded plant growth comparable with the level of the IC soil when treated with the bacterial inocula.

## Conclusions

The present study aimed at using an N-fixing bacterial species indigenous to Cr-loaded waste disposal sites which thus may have evolved metal detoxification mechanisms for biotransformation of Cr(VI) to non-toxic Cr(III) together with its potential to fix N. The gram-positive *Bacillus megaterium*-ASNF3 worked efficiently for detoxification of Cr-contaminated soil in the present investigation and reduced up to 86 % of 1000 mg/L of Cr(VI) in the soils within 60 days of incubation at 30 °C. Reduction mechanism was more active in liquid phase of the bioreactor as compared with the solid. The bacterium also retained its N-fixation potential up to 21.8 C_2_H_4_/mL/h while passing through exposure to toxic Cr and later bioremediation process. A soil rehabilitation success was achieved where wheat growths not only recovered in the treated soil but with even higher values of some growth parameters when compared with intact soils. These findings elucidate importance of the bacterium in agricultural as well as bioremedial technologies concomitantly.

## Abbreviations

C: carbon
Cr: chromium
CrC: chromium-spiked control
IC: intact control
C_2_H_4_: ethylene
N: nitrogen
N-ase: nitrogenase
O.D.: optical density
